# Defining language disorders in children and adolescents with Noonan Syndrome

**DOI:** 10.1002/mgg3.1069

**Published:** 2020-02-14

**Authors:** Giulia Lazzaro, Cristina Caciolo, Deny Menghini, Francesca Cumbo, Maria C. Digilio, Rossella Capolino, Giuseppe Zampino, Marco Tartaglia, Stefano Vicari, Paolo Alfieri

**Affiliations:** ^1^ Department of Neuroscience, Child and Adolescent Psychiatric Unit Bambino Gesù Children’s Hospital IRCCS Rome Italy; ^2^ Department of Human Science LUMSA University of Rome Rome Italy; ^3^ Department of Medical Genetics Bambino Gesù Children’s Hospital IRCCS Rome Italy; ^4^ Center for Rare Disease and Congenital Defects Fondazione Policlinico Universitario A. Gemelli Catholic University Rome Italy; ^5^ Genetics and Rare Diseases Research Division Bambino Gesù Children’s Hospital IRCCS Rome Italy; ^6^ Institute of Psychiatry Fondazione Policlinico Universitario A. Gemelli Catholic University Rome Italy

**Keywords:** IQ, Linguistic functioning, *PTPN11*, RASopathies, SLI

## Abstract

**Background:**

Noonan Syndrome is a developmental disorder characterized by a distinctive phenotype including facial dysmorphism, webbed neck, short stature, heart defects, and variable cognitive deficits as major features. Over the years, neuropsychological and behavioral studies explored alteration of cognitive functioning and related domains, such as learning, memory, and attention. To our knowledge, however, data concerning the language profile in this disorder is scarce. The aim of the present study was to detect specific language functioning combining nonverbal intelligence quotient and language abilities and to pinpoint strengths and weaknesses in the language domains.

**Methods:**

The language profile of 37 Italian participants with molecularly confirmed diagnosis of Noonan Syndrome was evaluated using specific tools to assess vocabulary and grammar comprehension and production, as well as phonological development.

**Results:**

We observed that 78% of affected individuals exhibited language impairment. Within language domains, the strong area was lexical production and grammar production was the weak area. Almost half the participants manifested a similar trend of specific language impairment. Nonverbal intelligence quotient only correlated with grammar comprehension.

**Conclusion:**

Our study expands present knowledge about the language profile in NS, and provides data that could enable more effective patient management and appropriate intervention.

## Graphical abstract

Little is known about the language profile in Noonan Syndrome. Current results revealed that almost 80% of children from our cohort presented language impairment. Specifically, an area of weakness could be linked to grammar production (52% of children exhibited a below‐average score); conversely, the strength of language profile seemed to be lexical production (81% of children obtained scores on average). The striking data were that almost 50% of individuals presented a “Specific Learning Impairment‐like” trend. Our findings did not mirror the distribution of Specific Learning Impairment in general population (~7%): thus, it could be read as a syndrome‐specific impairment. The present study also indicated that nvIQ appeared to be positively and moderately associated with grammar comprehension abilities.

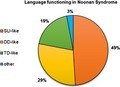

## 1 INTRODUCTION

Noonan syndrome (NS) is one of the most common nonchromosomal disorders affecting development (Noonan & Ehmke, 1963) with an estimated incidence of 1:1,000 to 1:2,500 live births (Mendez & Opitz, 1985). Major features include distinctive facial features (i.e., hypertelorism, ptosis, low‐set, and posteriorly rotated ears), short stature, a variable spectrum of heart defects, bleeding problems, lymphatic dysfunction, skeletal malformations, and variable cognitive deficits (Tartaglia, Gelb, & Zenker, 2011). NS belongs to a family of clinically related conditions collectively known as RASopathies, caused by mutations affecting signal transducers and modulatory proteins that play a role in the Ras‐mitogen‐activated protein kinase (RAS‐MAPK) pathway. Mutations affecting *PTPN11*, *SOS1*, *RAF1*, *RIT1,* and *LZTR1* account for the majority of NS cases (Aoki et al., 2013; Pandit et al., 2007; Roberts et al., 2007; Tartaglia et al., 2001, 2007).

The RAS‐MAPK pathway is a signal transduction cascade controlling a wide variety of cellular processes as well as early and late developmental processes, including brain development and function (Tartaglia & Gelb, 2010). Consistently, studies assessing the cognitive and behavioral profile of children with NS and other RASopathies have documented altered cognitive functioning (Wingbermühle et al., 2012), defective learning and memory (Alfieri, Cesarini, Mallardi, et al., 2011; Pierpont, Tworog‐Dube, & Roberts, 2013), attention (Pierpont, Tworog‐Dube, & Roberts, 2015) and visual processing (Alfieri, Cesarini, De Rose, et al., 2011; Alfieri et al., 2008). Moreover, altered sensory‐motor perception (Alfieri et al., 2008) and psychopathological profile (Alfieri et al., 2014; Perrino et al., 2018) have also been reported.

Despite recent efforts to characterize the cognitive and behavioral profile in NS and to explore genotype‐phenotype correlations, speech and language profile in this disorder have received little attention, and this field remains a challenge with regard to the neuropsychological characterization of this disorder. Hopkins‐Acos and Bunker (1979) were the first to describe speech and language features of a single child with NS. The three year‐old child exhibited language functioning significantly below that of children of comparable age in both receptive and expressive language. Nevertheless, despite the child's intellectual disability, the authors considered the delayed language development to be the result of a limited sensory‐motor experience associated with reduced motor development caused by concomitant congenital heart defect. Subsequently, Wilson and Dyson (1982) described a single case study of a seven year‐old female. The language profile was assessed in both expression and reception domains (morphological, semantic, pragmatic, and phonological). The authors reported a well‐developed communication despite a deviant phonological system, below‐average comprehension of single words and spatial and temporal concepts, and expressive morphological and syntactical skills below the expected level for her chronological age. Later studies (Cornish, 1996; Money & Kalus, 1979) have produced evidence indicating possible patterns of strengths and weaknesses in NS with a distinctive cognitive profile of good verbal fluency and vocabulary in contrast with poor nonverbal skills (e.g., visuo‐constructional skill). These studies suggested that NS is characterized by strengths in nonverbal skills but weakness in verbal skills, including those requiring comprehension and retention of verbal information. More recently, Wingbermühle and colleagues (2009) investigated the neuropsychological profile of NS to better understand how cognitive and behavior domains are influenced by genetic and neural makeup. The authors underlined the normal range of intelligence quotient (IQ) level during childhood despite impaired linguistic functions. Pierpont et al. (2010) examined the language profile and its association with other cognitive, motor, and perceptual factors in a large cohort of children and adolescents with NS (66 individuals, including 41 subjects with molecularly confirmed diagnosis). The study confirmed significant impairments in expressive and receptive language and severe difficulty with articulation. Furthermore, the authors documented a significant association between linguistic and cognitive functioning.

Based on these considerations, the object of our study was to characterize the language profile in a cohort of Italian children with molecularly confirmed diagnosis of NS. Specifically, the aim of the study was to detect specific trends combining nonverbal IQ (nvIQ) and language abilities, and to pinpoint strengths and weaknesses in the language domains. Based on our group's clinical experience, we could hypothesize that the language profile in NS is impaired despite the level of nonverbal cognitive functioning.

## 2 MATERIAL AND METHODS

### 2.1 Ethical compliance

Informed consent was obtained from all parents prior to participation and after receiving a comprehensive description of the study. The study was performed in accordance with the Declaration of Helsinki (1964) and was approved by the local ethical committee of the Bambino Gesù Children's Hospital.

### 2.2 Participants

Thirty‐seven Italian participants (13 females, 24 males) with molecularly confirmed diagnosis of NS (NS LAH in only one): 24 with *PTPN11* mutation, four with *RAF1* mutation, seven with *SOS1* mutation, one with *LZTR1* mutation, and one with *SHOC2* mutation (see Table 1) were recruited from the Child and Adolescent Psychiatry Unit and the Clinical Genetic Unit of the Bambino Gesù Clinical and Research Hospital in Rome. Age ranged from 3 to 13 years (mean age: 5.72; standard deviation [*SD*]: 2.25). Nonverbal intelligence quotient (nvIQ) ranged from 49 to 115 (mean nvIQ: 89.9; *SD*: 15). Audiologic histories revealed that six participants of 37 had a clinical history of hearing loss but only one required a hearing aid for normal daily activity (see Table 1). As part of this study, all individuals underwent a detailed neuropsychological evaluation to investigate cognitive and language profile.

**Table 1:** Characterization of our Italian cohort with molecularly confirmed diagnosis of NS/NS LAH

Subject	Gender	Years	Disorder	Gene	Amino acid change	Hearing problems
1	M	5.08	NS	*PTPN11*	Asn58Lys	–
2	M	3.83	NS	*PTPN11*	Pro491Leu	CHL
3	M	7.83	NS	*PTPN11*	Asn308Ser	–
4	M	3.75	NS	*PTPN11*	Glu139Asp	–
5	M	6.58	NS	*PTPN11*	Ala72Ser	–
6	M	6.75	NS	*PTPN11*	Asn308Ser	–
7	M	5.75	NS	*PTPN11*	Asp71Gly	–
8	M	4.91	NS	*PTPN11*	Leu262Arg	–
9	M	6.08	NS	*PTPN11*	Asn308Asp	–
10	M	5.91	NS	*PTPN11*	Asn308Asp	–
11	M	3.83	NS	*PTPN11*	Gln79Arg	–
12	M	3.66	NS	*PTPN11*	Thr52Ile	–
13	M	3.58	NS	*PTPN11*	Met504Val	–
14	F	6.41	NS	*PTPN11*	Phe285Ser	–
15	F	4.33	NS	*PTPN11*	Glu69Gln	Mild CHL
16	F	4.16	NS	*PTPN11*	Asn58Asp	–
17	F	7.33	NS	*PTPN11*	Asn308Asp	–
18	F	10.41	NS	*PTPN11*	Met508Val	–
19	F	4.58	NS	*PTPN11*	Arg498Trp	–
20	F	3.33	NS	*PTPN11*	Tyr63Cys	–
21	F	4.66	NS	*PTPN11*	Met504Val	–
22	F	6.25	NS	*PTPN11*	Asn308Ser	–
23	M	13.17	NS	*PTPN11*	Glu139Asp	SNHL
24	M	6.08	NS	*PTPN11*	Asn308Asp	–
25	F	4.25	NS	*RAF1*	Pro261Ala	Mild CHL (only right ear)
26	M	4.91	NS	*RAF1*	Pro261Leu	OM
27	F	5.50	NS	*RAF1*	Ser257Leu	Mild CHL
28	F	4.08	NS	*RAF1*	Asp486Gly	–
29	M	3.83	NS	*SOS1*	Arg552Gly	–
30	M	4.25	NS	*SOS1*	Met269Thr	–
31	M	5.50	NS	*SOS1*	Gly434Arg	–
32	M	5.33	NS	*SOS1*	Met269Thr	–
33	M	6.16	NS	*SOS1*	Tyr702His	–
34	F	5.66	NS	*SOS1*	Gln426Pro	–
35	M	7.17	NS	*SOS1*	Ile437Thr	–
36	M	4.16	NS	*LZTR1*	Arg478Trp	Mild CHL
37	M	12.42	NS LAH	*SHOC2*	Ser2Gly	–

Abbreviations: −, feature absent; CHL, conductive hearing loss; F, female; M, male; NS LAH Noonan Syndrome with loose anagen hair; NS, Noonan Syndrome; OM, otitis media; SNHL, sensorineural hearing loss.

### 2.3 Materials

#### 2.3.1 Nonverbal cognitive assessment

Nonverbal cognitive profile was assessed using the Leiter International Performance Scale (Leiter‐Revised or Leiter‐3) (Cornoldi, Giofrè, & Belacchi, 2016; Roid & Miller, 1997) for the majority of participants, and the Wechsler intelligence scale (Perceptual Reasoning Index of Wechsler Intelligence Scale for Children Fourth Edition, WISC – IV and Performance IQ of Wechsler Preschool and Primary Scale of Intelligence Third Edition, WPPSI‐III) (Orsini, Pezzuti, & Picone, 2012; Sannio Fancello & Cianchetti, 2008) in six participants.

#### 2.3.2 Language assessment

Lexical comprehension was assessed using the Phono‐Vocabulary Test (TFL, Test Fono‐Lessicale) (Marotta, Vicari, & Luci, 2007) for the majority of participants. TFL is normally used to assess both receptive and expressive language in preschool children. The receptive subtest contains four pictures (a picture target; a semantic distractor; a phonologic distractor; and a nonrelated distractor) for each table (45 in all). The examiner pronounces a word illustrating one of the four pictures on the table and asks the participant to choose the picture(s) that the word describes. The total score is converted into percentiles (pc). The Peabody Picture Vocabulary Test – PPVT (Dunn & Dunn, 1997) was applied to eight participants to evaluate their lexical comprehension. Here, the examiner pronounces a word describing one of four pictures shown and asks the participant to point to or say the number of the picture(s) that the word describes. The total score is converted into Lexical Quotient (LQ) and the pc computed.

TFL (Marotta et al., 2007) was used to assess lexical production in the majority of participants. The expression subtest contains the same pictures as the receptive subtest. The examiner points to a picture on the table and asks the participant to name the target picture. The Boston Naming Test (Kaplan, Goodglass, & Weintrab, 1983) was administered to evaluate lexical production to seven participants. The child is asked to tell the examiner the name of each picture and is given about 20 s to respond to each question. The total score was converted into *SD* and the pc calculated.

Grammar comprehension was investigated using the Grammar Evaluation Test (PVCL, Prove di Valutazione della Comprensione Linguistica) (Rustioni Metz Lancaster, 2007). Each test stimulus is presented in a four‐picture forced‐choice format. The examiner pronounces a sentence describing one of the four pictures and asks participants to choose the target picture(s). The total score was converted into seven levels of performance (nonsufficient; low; medium‐low; medium; medium‐high; high; very high) and the equivalent age (EA) was computed. To calculate EA, a score ranging from −1 to 1 was arbitrarily attributed depending on the level of performance obtained by the participants. Scores ranging from −1 to 0 were attributed when the range of performance obtained was respectively equal to or up to 1 year below their chronological age (CA). Conversely, a score ranging from 0.1 to 1 was attributed when the range of performance obtained was up to 1 year above their CA, Scores of −1, −0.66, −0.33, 0, 0.33, 0.66, and 1 were attributed to nonsufficient, low, medium‐low, medium, medium‐high, high, very high performance respectively. For instance, if a child 5.9 years old obtained a medium‐low score, the EA was computed as follows: 5.9 + (−0.33) = 5.57. Moreover, an index of deviation was calculated by subtracting CA from EA (Δ_grammar deviation_)_._

Sentence repetition could be considered as a measure of early grammatical development in Italian children (Devescovi & Caselli, 2007). A sentence repetition test is a reliable measure of the mean length of utterance that discriminates between different ages and identifies children with language problems (Devescovi & Caselli, 2007). Thus, the Test of Sentence Repetition (TSR)**,** a subtest of the Linguistic Evaluation Test (TVL, Test di Valutazione Linguistica) (Cianchetti & Sannio Fancello, 2003), was administered to investigate grammar production skills. The examiner pronounces a sentence and asks participants to repeat it. The raw score was converted into *SD* and the pc calculated.

#### 2.3.3 Speech assessment

##### Phonological development

A speech therapist evaluated phonological aspects through a collection of spontaneous utterance. We referred to the phonological development in the Italian language reported by Bortolini (2004) to determine the presence of delay, disorder or age appropriate phonology.

### 2.4 Analyses

First, mean (*SD*), median (min‐max) and confidence interval at 95% was calculated for age, nvIQ, language comprehension, and production measures.

Secondly, the percentage of participants who obtained a score below average (≤5th pc or Δ_grammar deviation_ ≤−1) in at least one language measure and/or showed evidence of a phonological disorder was calculated in order to estimate the proportion of language impairment in our sample. In addition, to examine the strengths and weakness of language skills in our cohort, scores of lexical/grammar production and lexical comprehension (considered as pc) and grammar comprehension (considered as Δ_grammar deviation_) were classified using a “traffic‐light system” with the following cut‐off (Croucher & Williamson, 2013; Holdnack et al., 2017): “red zone” (≤5th pc or Δ_grammar deviation_ ≤−1), “amber zone” (between 6th to 15th pc or −0.99 < Δ_grammar deviation_ ≤ −0.66), and “green zone” (>15th pc or Δ_grammar deviation_ > −0.66).

Thirdly, in order to determine the proportion of participants who exhibited a specific language impairment‐like (SLI‐like) behavior, our cohort was divided into three subgroups based on the nvIQ and language measures: 1. SLI‐like group – nvIQ ≥ 85; at least one language measure ≤5th pc or Δ_grammar deviation_ ≤ −1; 2. Developmental delay‐like (DD‐like) group – nvIQ < 85; at least one language measure ≤ 5th pc or Δ_grammar deviation_ ≤ −1; 3. Typical development‐like (TD‐like) group – nvIQ ≥ 85; all language measures > 5th or Δ_grammar deviation_ > −1.

Finally, Pearson's correlations between nvIQ and language measures were run. To ensure stability of the results, bootstraps were run (bootstrapping at 5,000 cycles). To control for multiple comparisons, the Bonferroni correction was applied (.05/4 language measures; critical *p*‐value of .01).

## 3 RESULTS

Descriptive analysis for nvIQ and language measures (lexical comprehension and production, grammar comprehension and production) of our cohort are depicted in Table 2.

**Table 2:** Descriptive statistics of age, nvIQ, and language measures

Measures	Mean	Range	*SD*	CI 95%
Descriptive statistics				
Age	5.72	3.33–13.17	2.25	1.82–2.92
nvIQ	89.89	49.00–115.00	14.96	12.16–19.42
Language measures				
Lexical comprehension	31.12	1.00–95.00	29.56	24.05–38.41
Lexical production	52.27	1.00–95.00	32.83	26.70–42.64
Grammar comprehension	5.11	3.50–9.40	1.31	1.07–1.70
Δ grammar deviation	−0.60	−8.00–1.59	1.65	1.34–2.14
Grammar production	23.04	1.00–92.00	26.97	20.97–36.48

Abbreviations: CI, Confidence Interval; nvIQ, non‐verbal Intelligence Quotient;* SD*, Standard deviation.

According to the criteria, in our sample 29 individuals of 37 (78%) were qualified as having language impairment. Specifically, 70% of our cohort showed a stand‐alone phonological disorder or a deficit across language domains.

**Figure 1:**
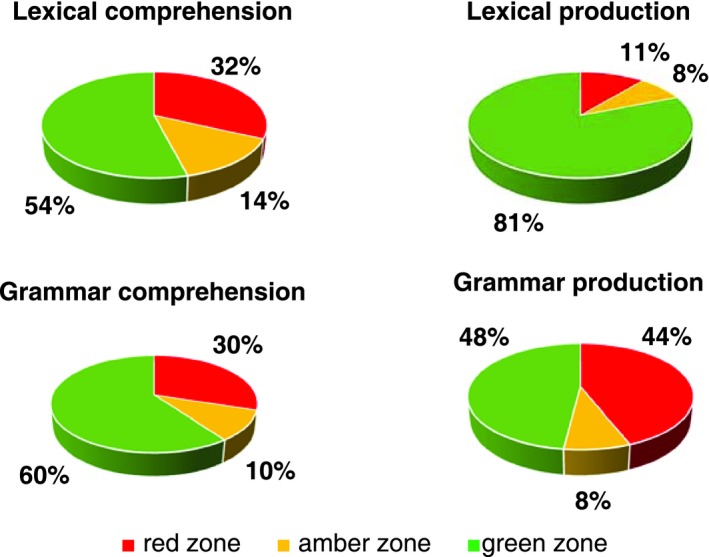
Proportion of the scores obtained by each participant in different language domains

Figure 1 shows, for different language domains, the percentage of the scores obtained by each participant in the group. Concerning lexical comprehension, all participants completed the tasks. 32% of the participants obtained a score within the “red zone”, 14% fell into the “amber zone”, and the rest 54% into the “green zone”. Considering lexical production, most of the participants (81%) fell into the “green zone” and only 11% into the “red zone”. As regards grammar comprehension, little more than half the participants (60%) obtained a score within the “green zone” and the 30% within the “red zone”.

Concerning grammar production, 27 of 37 participants completed the task. Almost half the participants (44%) fell into the “red zone”, while 48% fell into the “green zone”.

**Figure 2:**
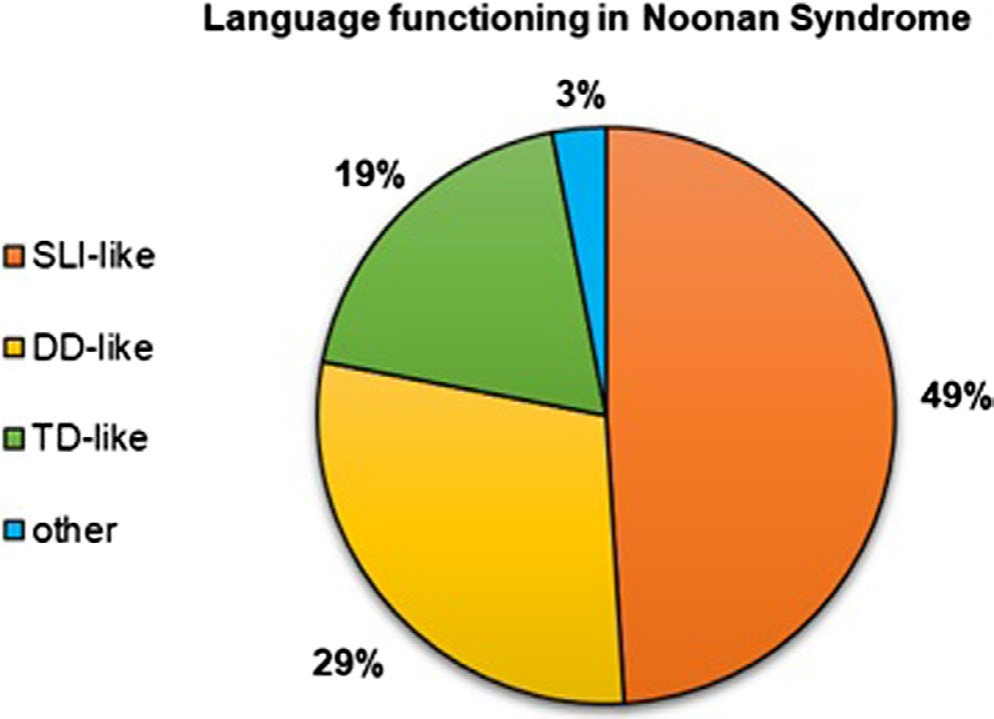
Percentage of different language profile in NS

Figure 2 illustrates the proportion of the three subgroups in the study cohort. Namely, in the group exhibiting a language or phonological impairment, almost half the participants (18 of 37) manifested a SLI‐like trend; of the other 50% (19 of 37), 29% exhibited a DD‐like behavior and 19% a TD‐like behavior. Finally, 1 child of 37 children had nvIQ < 85 and all language measures above average.

**Figure 3:**
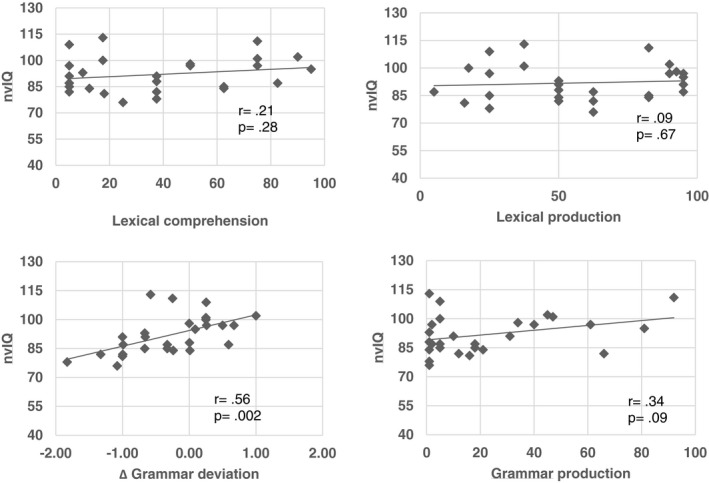
Correlations between nonverbal IQ and different language domains

As shown in Figure 3, nvIQ correlated significantly and positively with grammar comprehension (*r* = .56, CI = [.27, 0.81], *p* = .002) showing that higher nvIQ, larger Δ_grammar deviation_.

## 4 DISCUSSION

The aim of this research was to characterize the language profile in an unselected cohort of children with NS. To the best of our knowledge, this is the first study that attempts to delineate language profiling in a cohort of children with NS, all with a molecularly confirmed diagnosis.

Descriptive analyses were performed to detect a specific language functioning combining nvIQ and language abilities. First, we defined the proportion of children with NS who presented language impairment, such as a below‐average performance in at least one language and/or speech domain independently of the nvIQ. Results revealed that a high percentage of children from our cohort, almost 80%, presented language impairment. In accordance with the findings of Shah and colleagues (1999) and Romano and colleagues (2010), we also found that the NS group showed frequent speech problems (70%). An in‐depth analysis found that an area of weakness in language profile could be linked to grammar production, showing that 52% of children exhibited a below‐average score in TSR. Conversely, the strength of language profile in our cohort concerned lexical production domain showing that 81% of children obtained average scores.

Even considering only children who showed language impairment with average nvIQ, our findings did not mirror the distribution of SLI in the general population (~7%; Tomblin et al., 1997) since almost 50% of individuals in our cohort presented a “SLI‐like” trend: thus, it could be read as a syndrome‐specific impairment.

The present study also indicates that nvIQ appeared to be positively and moderately associated with grammar comprehension abilities. It suggested better performance in grammar comprehension, increased nvIQ. No other relation was seen between nvIQ and different language domains. Whether language function is commensurate with intelligence or shows a pattern of selective impairment is still debatable. Pierpont and colleagues (2010) revealed that language development and nonverbal cognitive ability in NS exhibits a “synchronous” pattern, similar to that observed in Fragile X syndrome (Abbeduto et al., 2003). Conversely, our findings are in line with findings in several syndromic conditions showing a pattern where the language profile appears more impaired than the cognitive one. For instance, research on Down syndrome has underlined that grammar abilities were more compromised than lexical ones (Galeote, Soto, Sebastián, Checa, & Sánchez‐Palacios, 2014). Further studies suggested that difficulties in grammar production were more impaired in children with Down syndrome than in those with typical development, even controlling for mental age (Caselli, Monaco, Trasciani, & Vicari, 2008; Vicari, Caselli, Gagliardi, Tonucci, & Volterra, 2002; Vicari, Caselli, & Tonucci, 2000). In addition, Barnett and van Bon (2015) described further chromosomal aberrations such as 1p21.3 deletion, 7q11.23 microduplication, and 15q11.2 deletion that are associated with speech and language pathology occurring in the setting of normal or only mildly impaired cognitive function.

The only study that attempted to add to present scientific knowledge about language phenotype in children and adolescents with NS was carried out by Pierpont and colleagues (2010). Our data, however, do not concur with this earlier study. First, they found that 30% of the cohort were qualified as having language impairment and 20% of the total sample fell in the range of significant articulation impairment. Another discrepancy is linked to the prevalence of children with “SLI‐like” functioning since the authors estimated the percentage of “SLI‐like” at around 5%, similar to the general population (~7%, Tomblin et al., 1997). Lastly, they found a strong correlation between nvIQ and the index of language abilities assessed by Clinical Evaluation of Language Fundamentals – Preschool, Second Edition (CELF‐P2) (Semel, Wiig, & Secord, 2004) or Clinical Evaluation of Language Fundamentals – Fourth Edition (CELF‐4) (Semel, Wiig, & Secord, 2003). A possible explanation for such discrepancies could be due to methodological dissimilarities. First, in our study only individuals with molecularly confirmed diagnosis were included. Also, our sample was characterized by a lower mean age (mean age: 5.72; *SD*: 2.25 vs. mean age 10.0, *SD*: 4.1). Secondly, in the present study we collected data for isolated language domains: comprehension and production were assessed for both lexical and grammar measures. Pierpont and colleagues (2010), however, used a relatively broad indicator of language functioning (CELF‐P2; CELF‐4). Nevertheless, despite such dissimilarities, the two samples showed similar nvIQ ranges and means.

Both studies contributed considerably to the characterization of language profile, and raised interesting questions about the impact of differences due to experimental design.

Given the great proportion of “SLI‐like” profiles found in a cohort of Italian NS, evidence‐based medicine should greatly assist clinicians to make a meticulous assessment, as well as therapists for intervention planning. Until now, there have been no studies focusing on “gold standard” interventions for language difficulties in NS. In the light of these considerations, early intervention is essential, and considered more valid than “watch and wait” (Glogowska, Roulstone, Enderby, & Peters, 2000). In addition, as discussed by Law and colleagues (2004), it would be useful to promote intensive interventions lasting at least eight weeks to obtain the best clinical outcome to support severe expressive deficits. One limitation of this study is the lack of a control group: our analyses focused on comparisons with normative data. With no control group, it would have been difficult to make power calculations. A measure for stability of statistical analysis was applied in order to compensate for this limitation. Future studies should be designed to include a control group and enlarge the sample size to obtain more consistent data and to better characterize the development of language in NS through longitudinal studies. A second limitation concerns the lack of observations of pragmatic functioning which, along with language skills, is a crucial ability for effective communication. Selås and Helland (2016) provided a preliminary characterization of communication and pragmatic profile in children with diagnosis of NS, even if not always molecularly confirmed. This study discussed a potential pragmatic impairment assessed using the Children's Communication Checklist, Second Edition (Bishop, 2011) in addition to language difficulties (in almost 77% of children in their cohort).

In conclusion, our study opens up novel possibilities for in depth investigation of the phenotypic manifestation of language in NS in order to improve clinical evaluation and to support appropriate intervention.

## CONFLICT OF INTEREST

The authors declare no conflict of interest.
